# *Salacia* spp.: recent insights on biotechnological interventions and future perspectives

**DOI:** 10.1007/s00253-023-12998-z

**Published:** 2024-02-08

**Authors:** Jaykumar Chavan, Priyanka Patil, Avdhoot Patil, Akshay Deshmukh, Pallavi Panari, Ashwini Mohite, Pramod Lawand, Pradnya Yadav, Minal Bodhe, Abhijit Kadam, Dada Namdas, Bandu Pawar, Amol Jadhav, Mahipal Shekhawat, Claudette Santa-Catarina

**Affiliations:** 1 Department of Botany, Yashavantrao Chavan Institute of Science (Autonomous), Lead College of Karmaveer Bhaurao Patil University, Satara, 415001 India; 2 Department of Microbiology, Yashavantrao Chavan Institute of Science (Autonomous), Lead College of Karmaveer Bhaurao Patil University, Satara, 415001 India; 3 Plant Biotechnology Unit, Kanchi Mamunivar Government Institute for Postgraduate Studies and Research, Puducherry, 605008 India; 4 Laboratório de Biologia Celular E Tecidual (LBCT), Centro de Biociências E Biotecnologia (CBB), Universidade Estadual Do Norte Fluminense Darcy Ribeiro (UENF), Av. Alberto Lamego 2000, Campos Dos Goytacazes, RJ 28013-602 Brazil

**Keywords:** *Salacia*, Tissue culture, Propagation, Microbial endophytes, Secondary metabolites, Molecular markers, Medicinal plants, Nanomaterials

## Abstract

**Abstract:**

The plants of the genus *Salacia* L. are the storehouse of several bioactive compounds, and are involved in treating human diseases and disorders. Hitherto, a number of reports have been published on in vitro biotechnology as well as microbial involvement in the improvement of *Salacia* spp. The present review provides comprehensive insights into biotechnological interventions such as tissue culture for plant propagation, in vitro cultures, and endophytic microbes for up-scaling the secondary metabolites and biological potential of *Salacia* spp. Other biotechnological interventions such as molecular markers and bio-nanomaterials for up-grading the prospective of *Salacia* spp. are also considered. The in vitro biotechnology of *Salacia* spp. is largely focused on plant regeneration, callus culture, cell suspension culture, somatic embryogenesis, and subsequent ex vitro establishment of the in vitro–raised plantlets. The compiled information on tissue cultural strategies, involvement of endophytes, molecular markers, and nanomaterials will assist the advanced research related to in vitro manipulation, domestication, and commercial cultivation of elite clones of *Salacia* spp*.* Moreover, the genetic diversity and other molecular-marker based assessments will aid in designing conservation policies as well as support upgrading and breeding initiatives for *Salacia* spp.

**Key points:**

*• Salacia spp. plays a multifaceted role in human health and disease management.*

*• Critical and updated assessment of tissue culture, endophytic microbes, metabolites, molecular markers, and bio-nanomaterials of Salacia spp.*

*• Key shortcomings and future research directions for Salacia biotechnology.*

## Introduction

**Fig. 1:**
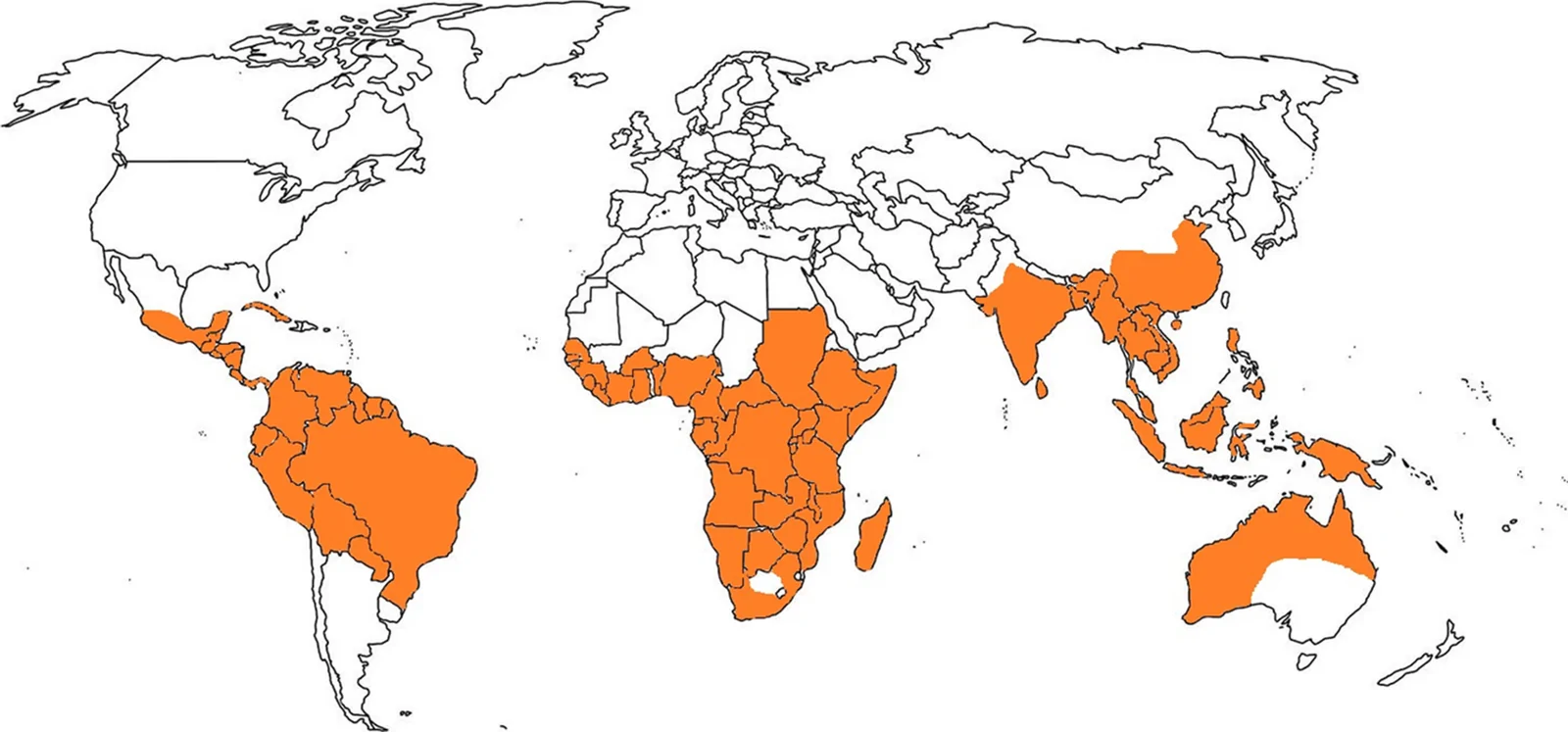
Worldwide distribution of *Salacia* spp

The medicinal and aromatic plants have immense value for the well-being of humans. These plants are not only valued for food but also serve as major natural resources for bioactive molecules and remedies against several diseases. Moreover, the natural extracts, surfactants, and viscous juice of aromatic and medicinal plants possess enormous catalytic influence during synthesizing pharmaceutically important chemicals (Sapkal et al. 2023). Genus *Salacia* L. (Celastraceae-Salacioideae) possesses medicinally important plants with worldwide distribution in their native range in the tropics and subtropics. Plants of the genus *Salacia* are climbing shrubs, small trees, or lianas. They consist of over 200 species, primarily distributed in the Indian Subcontinent, African countries, and torrid-zone areas such as Brazil (Nandikar 2021). The maximum diversity of the species is found in South Africa (covering over half of the total count), and the remaining species occur in the neotropics and tropical Asia. The distribution of the plants of the genus *Salacia* is presented in Fig. 1. Many species are routinely used in numerous traditional systems of medicine due to the occurrence of numerous bioactive compounds belonging to xanthones, terpenes, phenolics, glycosides, thiosugars, etc. (Chavan et al. 2015a; Morikawa et al. 2021; Chavan and Santa‑Catarina 2023). In recent years, dietary supplements and health foods formulated from *Salacia* plants have been attaining more admiration in Japan and other countries (Dubey et al. 2011). The morphological similarities between *Salacia* plants, particularly in the medicinally used parts (roots and stems), become a serious challenge in the well-regulated usage of *Salacia* health foods. However, recent advancements in plant biotechnology, especially molecular markers, assisted in identifying the adulteration of other plant species and authenticating *Salacia*-based health foods (Zhu et al. 2021).

Inadequate cultivation practices and indiscriminate collection of many *Salacia* species for supplementing universal requirements of herbal medicine becomes a hurdle for the survival of the natural populations of many *Salacia* spp. However, plant tissue culture and recent developments in plant-based endophyte provide insights into the involvement of these tools in modulating the biosynthesis of numerous industrially important phytochemicals, which helped to reduce pressure on natural resources. Interventions of in vitro biotechnology and endophyte-mediated secondary metabolite production open new avenues for bioprospecting the *Salacia* spp.

### Morphological attributes of *Salacia* spp.

**Fig. 2:**
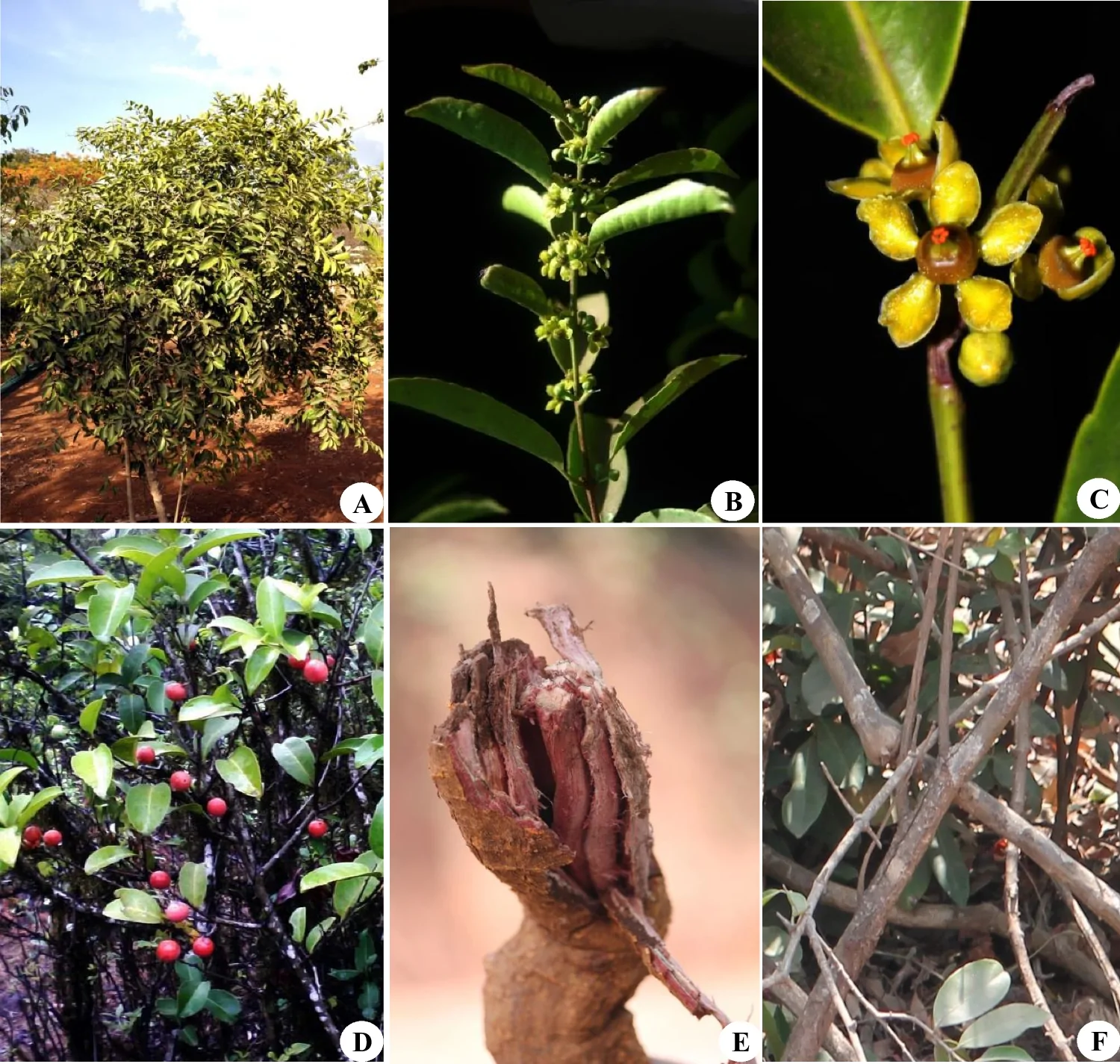
Phenotypic attributes of *Salacia* spp. **A** Habit of *S. chinensis*; **B** inflorescence of *S. chinensis*; **C** flower of *S. chinensis*; **D** fruits of *S. chinensis*; **E** root of *S. macrosperma*; **F** stem of *S. macrosperma*

*Salacia* species are scandent or sarmentosa shrub or small trees or climbing or strangling shrub. Stem color grayish to greenish, acute branching, and strangling stem. Leaves: elliptic or oblong, leaf blade shape narrowly ovate/round, leaf apex shape acute to acuminate, leaf base shape obtuse/wedge, or round or entire to crenulated leaf edges jagged/serrated, margins dentate, leaf blade shape elliptic/narrowly ovate. Flowers: inflorescence verticil, 3–6 flowers on axillary fascicles, petal 0.3 cm, pedicle 0.4–0.6 cm long, flower color yellowish green, stellate corolla, petals broadly ovate, sepal shape oblong or obovate or elliptic, sepal length up to 0.1 cm, anther thecae color orange, calyx lobe triangular, minute ovate to entire. Fruits: immature fruit color green, red when ripening, fruit 1–5 cm in diameter, fruit shape round/ovate, apex shape obtuse, fruit surface smooth/tuberculate/rugose, pericarp thin, flesh thickness thin, mesocarp pale yellow/light orange. Seeds: one-two seeded, shape round/oblong/ellipsoid color brown/light orange/gray. The phenotypic attributes of a few *Salacia* spp. are presented in Fig. 2.

### Traditional implications, phytochemistry, and biological properties

**Fig. 3:**
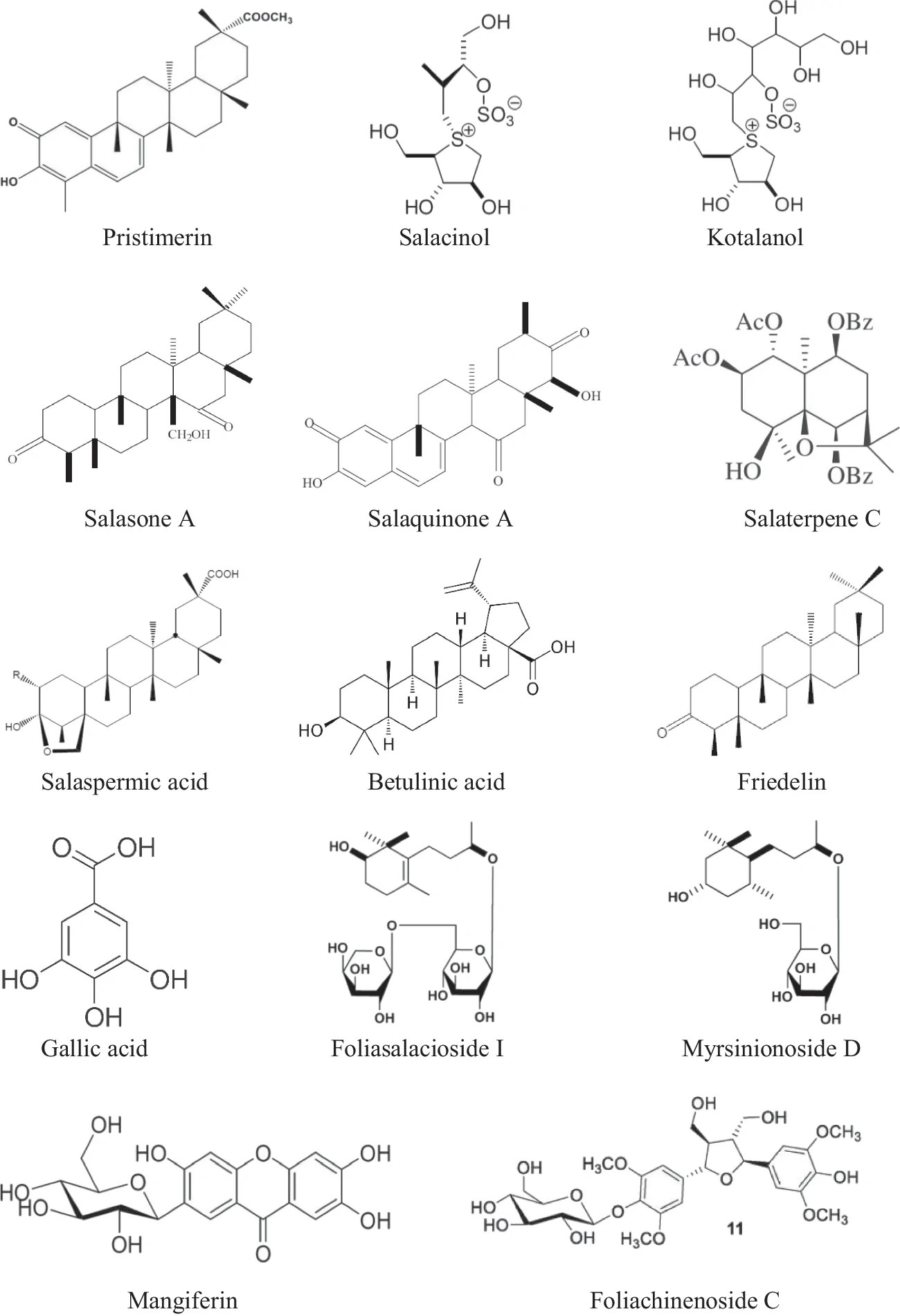
Noteworthy bioactive phytoconstituents of *Salacia* spp

Many *Salacia* spp. have proven their role as traditional remedies for different diseases and disorders in humans. The published reviews and original articles provide an account of the phytochemical composition and biological properties of *Salacia* spp. (Paarakh et al. 2008; Arunakumara and Subasinghe 2010; Stohs and Ray 2015; Kushwaha et al. 2016; Morikawa et al. 2021). These reports highlight the possible role of *Salacia* spp. in the traditional medicinal system, as these plants are practiced as acrid, bitter, thermogenic, for urinary infections, astringent, anodyne, anti-inflammatory, depurative, emmenagogue, vulnerary, liver tonic, and stomachic and many more. The crude extracts and the bioactive constituents isolated possess pharmacological properties including antioxidant, anti-obese, anticancer, antiproliferative, anti-HIV etc. and as remedies for Alzheimer’s and Parkinson’s diseases (Chavan et al. 2013; Feng et al. 2019). Moreover, different plant organs accumulate several phytochemicals that are routinely used in clinical practices (Ghadage et al. 2017; Chavan and Santa‑Catarina 2023). The key phytochemicals reported from various *Salacia* spp. are presented in Fig. 3.

### Conventional propagation strategies

Conventional propagation using vegetative organs such as roots, shoots, leaves, and seeds is among the safe and effective routes for preserving genetic authenticity and producing metabolite-rich clones of medicinally important plants. The published articles confirm root and stem cuttings as common conventional strategies for propagation of *Salacia* spp. Available literature confirms that scientific knowledge on vegetative propagation for the genus *Salacia* was initiated by Sasidharan et al. (2010) in *S. oblonga*. This study reported the requirement of 6000 ppm of indole-3-butyric acid (IBA) application on the stem cuttings of *S. oblonga*, which resulted in 80% rooting and a 60% plant survival rate. On the other hand, Rathnayaka and Subasinghe (2010) successfully employed a pruning strategy for enhanced growth of stems and leaves of *S. reticulata* following different treatments with biofertilizers. The results suggested that pruning at 50 cm height produces a significantly higher number of branches, and even more branches are developed when plants are fertigated with T_200_.

Nayana et al. (2015) assessed the efficacy of the maturity stage of stem cuttings and potting media on vegetative propagation of *S. reticulata*. Soft wood, semi-hard wood, and hard wood stem cuttings of *S. reticulata* were transferred to a potting mixture of top soil, and compost (1:1) to assess the effect of maturity stage on the rooting of stem cuttings. Moreover, different types and concentrations of potting substrate such as sand, top soil, coir dust, and compost were tested either alone or in their combinations. The findings confirmed that the mixture of top soil and compost (1:1) emerged as the optimal potting substrate for planting semi-hard stem cuttings of *S. reticulata*. The root and stem cuttings of *S. oblonga* were treated with diverse applications of IBA (0–500 ppm), wherein the stem with leaves showed the maximum shooting response at 300 ppm, and the roots showed the maximum response at 200 ppm (Deepak et al. 2015a). Muhammad Anaz et al. (2017) developed a stem cutting–mediated vegetative propagation technique for five *Salacia* spp. including *S. brunoniana*, *S. malabarica*, *S. oblonga*, *S. gambleana*, and *S. fruticosa*. Among the different IBA concentrations, 4000 ppm (*S. brunoniana*), 7000 ppm (*S. malabarica*), 7000 ppm (*S. oblonga*), 8000 ppm (*S. gambleana*), and 7000 ppm (*S. fruticosa*) served optimum for the induction of roots and subsequent plantlet establishment. Recently, Gunaga and Vasudeva (2019) successfully employed the air-layering technique for the propagation of the threatened and potential anti-diabetic plant, *Salacia macrosperma*.

### Tissue culture and in vitro propagation of* Salacia *spp*.*

**Fig. 4:**
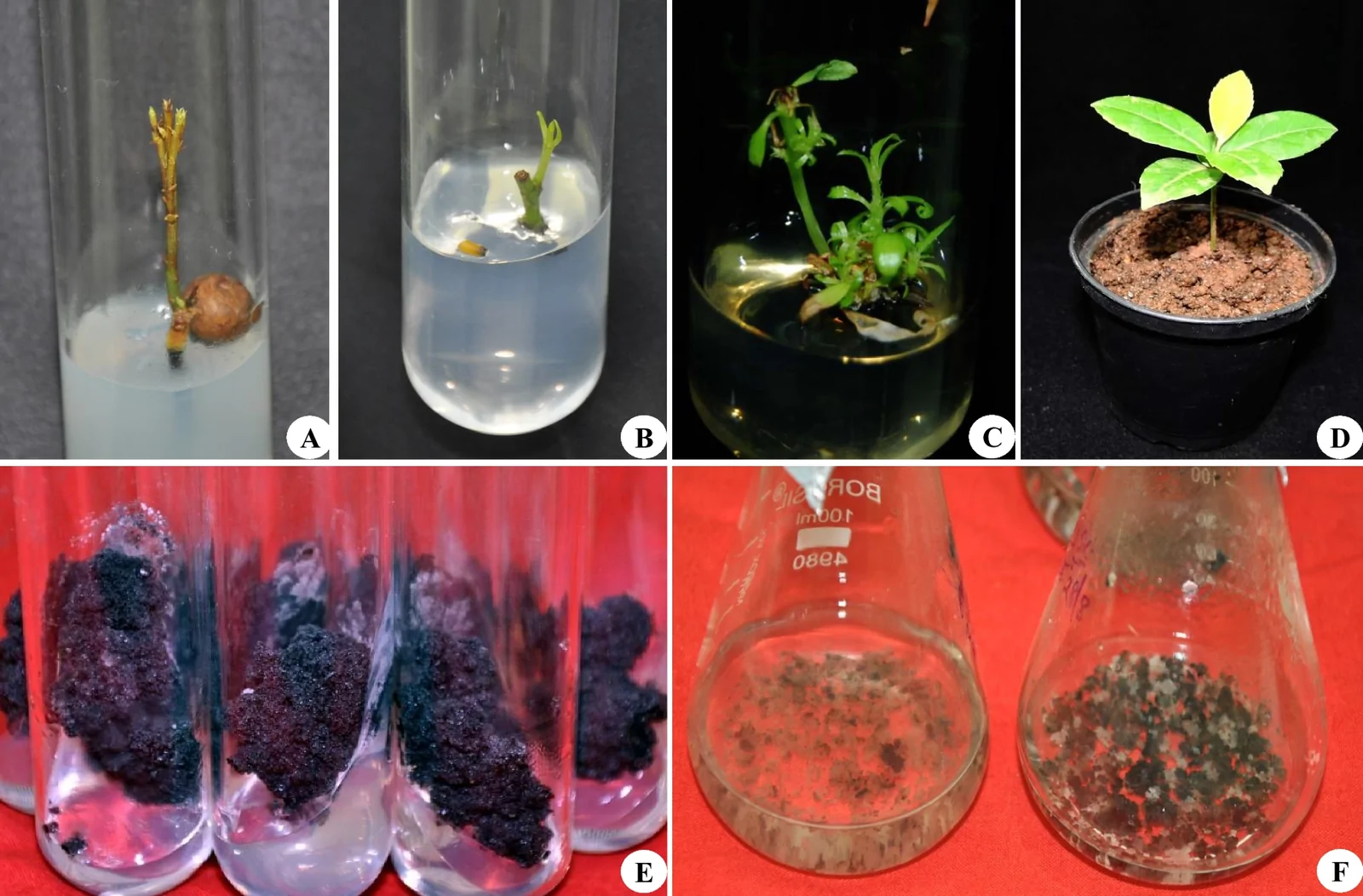
Tissue culture studies of *S. chinensis.* **A** Seed germination; **B** shoot induction from nodal explant; **C** shoot multiplication (in Chavan et al. 2015b); **D** hardening (in Chavan et al. 2015b); **E** callus proliferation; **F** cell suspension culture

Plant tissue culture is a crucial biotechnological approach for large-scale propagation and subsequent conservation of endemic, endangered, aromatic, medicinal, and wild relatives of crop plants (Chavan et al. 2018a; Babar et al. 2020; Kaur et al., 2021; Chavan and Dey 2023). Micropropagation, somatic embryogenesis, callus culture, cell suspension culture, thin cell layer, synthetic seeds, and in vitro organ cultures are among the routinely employed tissue-culture strategies for propagation, conservation, secondary metabolite production, and improvement of the agronomical traits of several medicinal plants. These in vitro strategies are reliable, rapid, cost-effective, and able to produce a paramount number of saplings in a limited time duration. Nowadays, the trend has shifted toward large-scale production and obtaining medicinal plants with elite attributes of commercial importance using tissue culture-based propagation strategies (Kshirsagar et al. 2021; Sanyal et al. 2023a). *Salacia* spp. are propagated using various in vitro propagation systems for different purposes. Table 1 represents the various in vitro regeneration approaches for *Salacia* spp. Moreover, the in vitro regeneration stages of *S. chinensis* are presented in Fig. 4.

**Table 1:** Experimental details and various in vitro morphogenetic responses of *Salacia* spp

*Salacia* spp*.*	Morphogenetic response	Explant	Disinfection procedure	Optimal culture medium	Culture conditions	Experimental outcome	References
*S. reticulata*	Shoot multiplication	Nodes	TW, Teepol, NaOCl, 70% alcohol, 0.1% HgCl_2_	MS + BA (3.5 mg/l) + IAA (0.5 mg/l)	T: 25 ± 2 °C L: 60 µmol m^−2^ s^−1^ RH: 65%	An average of 10.64 ± 0.96 shoots/explant	Dhanasri et al. 2013
*S. reticulata*	Rooting	Shoots (in vitro)	–	Half-MS + IBA (2.0 mg/l)	T: 25 ± 2 °C L: 60 µmol m^−2^ s^−1^ RH: 65%	An average 3.05 ± 1.55 roots/shoot	Dhanasri et al. 2013
*S. chinensis*	–	Leaf	TW, dishwasher gel (5 min), SDW (5 times), 70% alcohol (2 min), 1% HgCl_2_ (2 min)	MS medium	T: 25 ± 2 °C L: 125 µmol m^−2^ s^−1^ RH: 55%	96% survival of nodal explants	Majid et al. 2014
*S. chinensis*	Shoot multiplication	Shoot segments (nodes)	TW, Labogent (20%, 10 min), HgCl_2_ (0.1%, 7 min), SDW	MS + BAP (2.0 mg/l) + NAA (0.8 mg/l) + AA (100 mg/l)	T: 25 ± 1 °C L: 40 µmol m^−2^ s^−1^	75% of shoot multiplication frequency with 6.7 ± 1.0 shoots/node	Chavan et al. 2015b
*S. chinensis*	*Rooting*	Shoots (in vitro)	–	½MS + IBA (1.5 mg/l)	T: 25 ± 1 °C L: 40 µmol m^−2^ s^−1^	88% rooting with 5.3 ± 0.2 roots	Chavan et al. 2015b
*S. chinensis*	Callus culture	Leaf (in vitro)	–	MS + 2,4-D (2.0 mg/l) + BAP (1.5 mg/l)	T: 25 ± 1 °C L: 40 µmol m^−2^ s^−1^	92% of callus induction frequency	Chavan et al. 2015b
*S. chinensis*	Shoot multiplication	Leaf, node, shoot tips	TW, dishwasher gel (5 min), 70% EtOH (2 min), 1.0% NaOCl + 2–3 drops of Tween-20 (15 min), SDW	MS + BAP (1.0 mg/l) + NAA (0.5 mg/l)	T: 25 ± 2 °C L: 125 µmol m^−2^ s^−1^ RH: 55%	87.81 ± 3.22% shoot proliferation with 5.37 ± 0.02 shoots/explant	Majid et al. 2016
*S. chinensis*	Rooting	Shoots (in vitro)	–	Half-MS + IBA (2.0 mg/l)	T: 25 ± 2 °C L: 125 µmol m^−2^ s^−1^ RH: 55%	91.33 ± 2.02% of root formation frequency with 4.35 ± 0.03 roots/shoot	Majid et al. 2016
*S. chinensis*	Callus formation	Leaf, nodes, shoot tips	TW, dishwasher gel (5 min), 70% EtOH (2 min), 1.0% NaOCl + 2–3 drops of Tween-20 (15 min), SDW	MS + NAA (1.0 mg/l) + BAP (2.0 mg/l)	T: 25 ± 2 °C L: 40 µmol m^−2^ s^−1^	93.43 ± 2.75% callus induction from nodes	Majid et al. 2017
*S. chinensis*	Shoot multiplication	Nodes	TW, dishwasher gel (5 min), 70% EtOH (2 min), 1.0% NaOCl + 2–3 drops of Tween-20 (15 min), SDW	MS + BAP (1.5 mg/l) + NAA (1.0 mg/l)	T: 25 ± 2 °C L: 40 µmol m^−2^ s^−1^	93.33 ± 2.02% shoot induction with 5.12 ± 0.09 shoots/explant	Majid et al. 2017
*S. chinensis*	Rooting	Shoots (in vitro)	–	Half-MS + IBA (2.0 mg/l)		91.66 ± 2.33% root induction frequency	Majid et al. 2017
*S. chinensis*	Shoot formation for elevating biological potential	Nodes	–	MS + NAA (0.5 mg/l) + BAP (1.0 mg/l)	T: 25 ± 2 °C L: 40 µmol m^−2^ s^−1^	Regenerated shoots were assessed for antioxidant and anti-diabetic properties	Majid et al. 2018
*S. chinensis*	Rooting	Shoots (in vitro)	–	Half-MS + IBA (2.0 mg/l)	T: 25 ± 2 °C L: 40 µmol m^−2^ s^−1^	–	Majid et al. 2018
*S. chinensis*	Shoot formation	Nodes	TW (30 min), Teepol (15 min), TW, 70% EtOH (1–2 min), SDW, 0.1% HgCl_2_ (5 min)	MS + BAP (2.0 mg/l) + NAA (0.8 mg/l)	T: 25 ± 2 °C L: 50 µmol m^−2^ s^−1^	An average of 3.90 ± 0.06 shoots	Laxmi et al. 2018
*S. chinensis*	Callus biomass production for metabolite accumulation	Leaf	TW, Labogent (0.5%, 5 min), 0.1% HgCl_2_ (3 min), SDW (3 times)	MS + BAP (2.0 mg/l) + NAA (0.8 mg/l) + JA (75 μM)	T: 25 ± 5 °C L: 40 µmol m^−2^ s^−1^	8.10 ± 0.6 g of calli biomass. Assessment of calli for secondary metabolites	Chavan et al. 2021
*S. chinensis*	Callus formation	Leaf segment	TW, dishwasher gel, ddH_2_O (5 min), 70% EtOH (2 min), 1.0% NaOCl + Tween-20, sterile ddH_2_O (4 times)	MS + TDZ (0.5 mg/l)	T: 25 ± 2 °C RH: 55%	100% callus induction	Kamat et al. 2021
*S. chinensis*	Shoot formation	Nodes	TW, dishwasher gel, ddH_2_O (5 min), 70% EtOH (2 min), 0.1% HgCl_2_, sterile ddH_2_O (4 times)	MS + BAP (3.5 mg/l) + IBA (1.0 mg/l)	T: 25 ± 2 °C RH: 55%	Shoot formation in 78.3% cultures	Kamat et al. 2021
*S. chinensis*	Shoot formation	Callus	–	MS + NAA (1.0 mg/l) + BAP (0.5 mg/l)	T: 25 ± 2 °C RH: 55%	85% shoot regeneration response with 12.33 ± 0.33 shoots/callus	Kamat et al. 2021
*S. chinensis*	Shoot formation	Seeds	TW, dishwasher gel, ddH_2_O (5 min), 70% EtOH (2 min), 2% NaOCl (15 min) sterile ddH_2_O (4 times)	MS + BAP (3.0 mg/l) + 2iP (2.0 mg/l)	T: 25 ± 2 °C RH: 55%	12.3 ± 0.40 shoots/explant	Kamat et al. 2021
*S. chinensis*	Rooting	Shoots (in vitro)	–	MS + IAA (0.5 mg/l)	T: 25 ± 2 °C RH: 55%	Root formation in 70 ± 1.3% cultures with 8.5 ± 0.28 roots/shoot	Kamat et al. 2021
*S. chinensis*	Callus production for augmenting antibacterial potential	Leaf	TW, Labogent (0.5%, 5 min), 0.1% HgCl_2_ (3 min), SDW (3 times)	MS + BAP (2.0 mg/l) + NAA (0.8 mg/l) + JA (75 μM)	T: 25 ± 5 °C L: 40 µmol m^−2^ s^−1^	Calli with antibacterial properties against seven laboratory pathogens	Chavan et al. 2022
*S. macrosperma*	Callus culture for enhancing biological potential	Leaf	TW, Bavistin (5%, 10 min), Tween-20 (10%, 2 min), 0.1% HgCl_2_ (3 min), SDW (3 times)	MS + 2,4-D (2.5 mg/l) + BAP (1.5 mg/l)	T: 21 ± 1 °C L: 3000 lx RH: 80%	98.33% of callus induction frequency and calli evaluated for polyphenol content, antimicrobial and antioxidant properties	Mahendra et al. 2020b
*S. macrosperma*	Callus formation	Leaf, nodes	TW, 0.2% Tween-20 (5 min), SDW, 1% Bavistin (10 min), SDW, 0.1% HgCl_2_ (3–4 min), SDW (3–4 times)	MS + 2,4-D (2.5 mg/l) + BAP (1.5 mg/l)	T: 21 ± 1 °C RH: 80%	Leaf explants produced the highest percentage of calli (98.33%)	Mahendra et al. 2020a
*S. macrosperma*	Shoot formation	Nodes	TW, 0.2% Tween-20 (5 min), SDW, 1% Bavistin (10 min), SDW, 0.1% HgCl_2_ (3–4 min), SDW (3–4 times)	MS + BAP (1.0 mg/l) + NAA (0.5 mg/l) + TDZ (0.5 mg/l)	T: 21 ± 1 °C RH: 80%	An average of 13.00 ± 0.57 shoots/node	Mahendra et al. 2020a
*S. macrosperma*	Rooting	Shoots (in vitro)	–	MS + IAA (1.0 mg/l)	T: 21 ± 1 °C RH: 80%	An average of 4.33 ± 0.88 roots/shoot	Mahendra et al. 2020a
*S. macrosperma*	Cell suspension culture	Callus	–	MS liquid	T: 21 ± 1 °C RH: 80%	Formation of 60% pro-embryos	Mahendra et al. 2020a
*S. oblonga*	Shoot formation	Node	–	MS + BAP	–	–	Sasidharan et al. 2010
*S. oblonga*	Shoot multiplication	Nodes	Detergent, 0.5% Bavistin (5 min), 0.1% HgCl_2_ (1 min), SDW (5–6 times)	MS + BAP (3.5 mg/l) + IBA (1 mg/l)	T: 25 ± 2 °C L: 3000 lx RH: 50–60%	Development of 50% shoot primordia	Deepak et al. 2015a, b
*S. oblonga*	Rooting	Shoots (in vitro)	–	MS + IBA (0.5 mg/l)	T: 25 ± 2 °C L: 3000 lx RH: 50–60%	–	Deepak et al. 2015a, b

*2iP* isopentenyl adenine; *2,4-D* 2,4-dichlorophenoxyacetic acid; *AA* ascorbic acid; *BAP* 6-benzylaminopurine (syn. BA); *ddH*_*2*_*O* double distilled water; *EtOH* ethanol, *HgCl*_*2*_ mercuric chloride, *IAA* indole-3-acetic acid, *IBA* indole-3 butyric acid, *JA* jasmonic acid, *L* light, *MS* Murashige and Skoog’s medium, *NAA* α-naphthalene acetic acid, *NaOCl* sodium hypochlorite, *RH* relative humidity, *SDW* sterile distilled water, *T* temperature, *TDZ* thidiazuron, *TW* tap water

The selection of explants and their surface disinfection have prime importance in plant tissue culture techniques . An attempt at in vitro culture establishment in *Salacia* spp. was made using nodal segments, especially in the cultures that were initiated for shoot multiplication and propagation purposes (Sasidharan et al. 2010; Dhanasri et al. 2013; Chavan et al. 2015b; Deepak et al. 2015b; Majid et al. 2017, 2018; Laxmi et al. 2018; Kamat et al. 2021; Mahendra et al. 2020a). However, diverse surface sterilization procedures have been employed for different *Salacia* spp. Most of the nodal explants cleaned undergoing tap water wash followed by immersion in different types of detergents. Mercuric chloride is a monotonous surface disinfectant used for sterilizing the nodal explants of *S. chinensis* (Majid et al. 2014; Chavan et al. 2015b; Laxmi et al. 2018; Kamat et al. 2021), *S. macrosperma* (Mahendra et al. 2020a), and *S. oblonga* (Deepak et al. 2015b), wherein sodium hypochloride has been used to disinfect nodal segments of *S. reticulata* (Dhanasri et al. 2013) and *S. chinensis* (Majid et al. 2016, 2017). Shoot tip explants have also been tested for their efficiency during establishing in vitro cultures of *S. chinensis* (Majid et al. 2016, 2017), whereas in both attempts, the shoot tips were surface sterilized with sodium hypochloride. Kamat et al. (2021) established the in vitro cultures of *S. chinensis* through sodium hypochloride–treated seed explants. Callus cultures of most of the *Salacia* spp. have been initiated using leaf explants (Chavan et al. 2015b, 2021, 2022; Majid et al. 2017; Mahendra et al. 2020a, b; Kamat et al. 2021), with a few exceptions such as node and shoot tip explants (Majid et al. 2017; Mahendra et al. 2020a).

Murashige and Skoog’s (MS) medium have been unanimously used for shoot induction and multiplication for almost all studied *Salacia* spp.; however, the requirements of distinct class and concentrations of plant growth regulators (PGRs) are found to be species specific. The first attempt at shoot formation has been reported for *S. reticulata* by Dhanasri et al. (2013). Among the distinct types and concentrations of PGRs tested, MS medium supplemented with a mixture of benzyl adenine (BA) (3.5 mg/l) and indole-3-acetic acid (IAA) (0.5 mg/l) was found to produce 10.64 ± 0.96 shoots per explant. For *S. chinensis*, nodes cultured on MS medium with 2.0 mg/l of BAP, 0.8 mg/l of α-naphthalene acetic acid (NAA), and 100 mg/l of ascorbic acid (AA) were found suitable for the production of shoots (6.7 ± 1.0) in 75% cultures (Chavan et al. 2015b). Majid et al. (2016) were able to produce 5.37 ± 0.02 shoots per explant in 87.81 ± 3.22% cultures when nodal buds were transferred to MS medium augmented with BAP (1.0 mg/l) and NAA (0.5 mg/l). In another attempt, the same research group tested elevated concentrations of BAP and NAA for shoot multiplication in *S. chinensis* (Majid et al. 2017), wherein maximum shoot induction frequency (93.33 ± 2.02%) with 5.12 ± 0.09 was accomplished when nodal buds were cultured on BAP (1.5 mg/l) and NAA (1.0 mg/l) enriched MS medium. In further studies, Majid et al. (2018) employed a similar protocol for the production of shoots in *S. chinensis* and assessed them for their anticancer and anti-diabetic potential. Further research on *S. chinensis* also confirmed the requirement of MS medium alongside BAP (2.0 mg/l) and NAA (0.8 mg/l) for shoot proliferation (Laxmi et al. 2018). Kamat et al. (2021) noted the highest shoot multiplication frequency for *S. chinensis* in MS medium supplemented with MS medium with BAP (3.5 mg/l) and IBA (1 mg/l). However, shoot multiplication from seed explants of *S. chinensis* was dependent on the addition of 2iP (2.0 mg/l) and BAP (3.0 mg/l) in MS medium (Kamat et al. 2021). A mixture of BAP (1.0 mg/l), NAA (0.5 mg/l), and TDZ (0.5 mg/l) was found suitable for maximum shoot production in *S. macrosperma* (Mahendra et al. 2020a), wherein an average of 13.00 ± 0.57 shoots per explant have been produced. MS medium mixed with BAP (3.5 mg/l) and IBA (1 mg/l) was found suitable for shoot multiplication in *S. oblonga* (Deepak et al. 2015b).

Callus cultures provide a platform for indirect plant regeneration as well as enhanced secondary metabolite accumulation. For the genus *Salacia*, the first attempt at callus culture has been reported for *S. chinensis* (Chavan et al. 2015b). The results confirmed the addition of 2,4-D (2.0 mg/l) and BAP (1.5 mg/l) in MS medium for leaf-derived calli induction and proliferation calli (92% of callus induction frequency). In subsequent studies, Majid et al. (2017) achieved over 93.43 ± 2.75% callus induction frequency in *S. chinensis* when explants inoculated on MS medium supplemented with NAA (1.0 mg/l) and BAP (2.0 mg/l). Chavan et al. (2021) employed MS medium alongside BAP (2.0 mg/l), NAA (0.8 mg/l), and JA (75 μM) for calli biomass production from the leaves of *S. chinensis*. The calli biomass was further assessed for its phytochemical composition and antioxidant potential. The same procedure has been practiced by Chavan et al. (2022) for calli production to assess their antibacterial properties. In contrast, MS medium with exclusive supplementation of TDZ (0.5 mg/l) was found suitable for leaf segment–derived callus induction in 100% cultures of *S. chinensis* (Kamat et al. 2021). The calli were further practiced for their indirect shoot regeneration efficiency, where 12.33 ± 0.33 shoots per calli explant were recorded in 85% cultures (Kamat et al. 2021). Mahendra et al. (2020b) achieved callus production in 98.33% of culture vessels when leaf segments of *S. macrosperma* were transferred to MS medium with 2,4-D (2.5 mg/l) and BAP (1.5 mg/l). The same observation was recorded in further studies of *S. macrosperma* (Mahendra et al. 2020a). The pro-embryos were developed in 60% liquid cultures from leaf-derived calli.

In general, rooting of in vitro–generated shoots largely depends on media type and strength, PGR concentrations and combinations, plant genotype, incubation conditions, etc. For the genus *Salacia*, different concentrations and combinations of auxins and cytokinins have been tested for in vitro rooting; however, it was observed that MS medium fortified with either IBA or IAA served better for root induction in *Salacia* spp. It was also noted that there was no induction of roots in a PGR-free medium. Dhanasri et al. (2013) reported the best root induction frequency when in vitro–raised shoots of *S. reticulata* were transferred to half-strength MS medium supplemented with IBA (2.0 mg/l). In this media composition, 3.05 ± 1.55 roots have been developed. Likewise, the shoots of *S. chinensis* rooted best in IBA (1.5 mg/l) enriched half-strength MS medium, wherein rooting was observed in 88% of cultures and 5.3 ± 0.2 roots were produced per shoot (Chavan et al. 2015b). In subsequent studies on *S. chinensis*, the half-strength MS medium with IBA (2.0 mg/l) served better for in vitro root induction (Majid et al. 2016, 2017, 2018). In contrast, Kamat et al. (2021) confirmed the utility of IAA (0.5 mg/l) instead of IBA for successful in vitro rooting in *S. chinensis*. In vitro rooting in microshoots of *S. oblonga* was achieved by the incorporation of IBA (0.5 mg/l) in MS medium (Deepak et al. 2015b). Moreover, microshoots of *S. macrosperma* also rooted best (4.33 ± 0.88 roots/shoot) in MS medium supplemented with IAA at 1.0 mg/l of concentration (Mahendra et al. 2020a).

The efficiency of different planting substrates has been assessed during the ex vitro establishment of in vitro–raised plantlets of different *Salacia* spp. In vitro–raised plantlets of *S. reticulata* with well-developed shoots and roots were transferred to a mixture of soil and vermicompost (2:1) for hardening and acclimation (Dhanasri et al. 2013). Garden soil, river sand, and coco peat (1:1:1) was served optimum during the ex vitro establishment of *S. chinensis* plantlets, wherein over 80% survival rate of plantlets has been recorded (Chavan et al. 2015b). However, Majid et al. (2016) transplanted the in vitro–derived plantlets of *S. chinensis* into plastic pots with sterile compost consisting of organic fertilizer, sand, and peat (2:2:1) and maintained them at 18–28 °C with relative humidity ranging from 75 to 90%. Over 87% of survivability in field conditions was recorded. In subsequent studies from the same research group, a similar composition of planting substrate served better during the hardening of *S. chinensis* plantlets (Majid et al. 2017). On the other hand, sand, soil, and vermiculite (1:1:1) supported the ex vitro growth of *S. chinensis* plantlets (Kamat et al. 2021); however, a vermiculite and perlite mixture (1:1) has been required during the hardening of *S. macrosperma* plantlets with 80% survivability (Mahendra et al. 2020a). A mixture of soil and vermiculate (1:1) supported the acclimatization of in vitro–raised plantlets of *S. oblonga* (Deepak et al. 2015b).

In general, the tissue culture and in vitro regeneration of different *Salacia* spp. require MS medium during different regeneration stages. The shoot formation was significantly influenced by PGRs; however, BAP and 2,4-D play crucial roles during shoot multiplication. In vitro rooting has been totally dependent on the incorporation of either IBA or IAA in half-strength MS medium, wherein IBA showed superiority over IAA. Sand and soil are the common planting substrates required during the ex vitro establishment of tissue culture–raised plantlets of different *Salacia* spp.

### In vitro cultures and endophytic microbes in elevating secondary metabolite production and bioactivities

Involvement of diverse types of in vitro culture systems is among the regular exercises for augmenting secondary metabolite yield in several medicinally important plants. The shoots, roots, callus, and a mixture of roots and shoots obtained in vitro, and roots from micropropagated plants and roots from field-grown plants of *S. chinensis* have been assessed for mangiferin content (Chavan et al. 2015b). The results confirmed the higher accumulation of mangiferin (593.87 ppm) in the callus cultures of *S. chinensis* on MS medium supplied with 2,4-D (2.0 mg/l) and BAP (1.5 mg/l). Similarly, different in vitro regeneration stages significantly alter the metabolite profile of numerous medicinal and aromatic plants (Chavan et al. 2018b). Majid et al. (2018) evaluated the micropropagated plantlets of *S. chinensis* for their antioxidant and anti-diabetic properties using DPPH, FRAP, α-amylase, and α-glucosidase assays. The experimental outcome confirmed the superiority of micropropagated plantlets over field-grown plantlets with respect to their antioxidant and anti-diabetic potential.

A recent study on elicitation confirmed the augmentation of calli biomass, polyphenolics, mangiferin yield, and antioxidant properties of *S. chinensis* callus cultures (Chavan et al. 2021). The calli established on MS medium supplemented with 2,4-D (2.0 mg/l), BAP (1.5 mg/l), and treated with jasmonic acid (75 µM) served best for achieving the highest calli biomass (8.10 ± 0.6 g of fresh weight), the content of total phenolics (68.49 ± 0.90 mg of gallic acid equivalent per grams of dry weight), flavonoids (26.18 ± 0.35 mg of quercetin equivalent per gram of dry weight), and mangiferin content (8.493 ± 0.193 mg per gram of dry weight). The same elicitor treatment also augmented the antioxidant potential of calli when analyzed using DPPH, FRAP, and metal chelating assays. In another study, Chavan et al. (2022) reported that the same media composition and elicitor treatment enhanced the antibacterial potential of calli against *Proteus vulgaris*. In contrast, the antimicrobial potential has been reported to be higher in the leaves of field-grown plants than the in vitro–derived calli of *S. macrosperma* (Mahendra et al. 2020b). The leaf extract is known to possess strong antibacterial activity against *E. coli* (15.50 mm), *Salmonella typhi* (17.33 mm), *Staphylococcus aureus* (15.50 mm), *Bacillus subtilis* (17.16 mm), and *Pseudomonas aeruginosa* (16.66 mm). Kamat et al. (2021) evaluated the in vitro–raised leaves and calli of *S. chinensis* for their phytochemical composition. Gas chromatography–mass spectrophotometric analysis detected 16 and 11 phytochemicals in calli and leaves, respectively. Moreover, calli also possessed an almost fivefold higher amount of mangiferin (26 mg/ml) as compared to leaves (4 mg/ml).

Endophytes are the microbes that reside at least a part of their life cycle in plants and animals without causing any harmful symptoms. Several medicinal and aromatic plant species have been used as complementary forms of medicine since ancient times. Current research on the bioprospecting of endophytes for secondary metabolite production confirms the highest priority in the scientific world (Sanyal et al. 2023b). The work on entophyte isolation, characterization, and assessment of their pharmacological potential for *Salacia* spp. has begun since the last decade (Table 2). Bhagya et al. (2011) isolated a fungal endophyte, *Colletotrichum gloeosporioides* Penz, from the stem and leaves of *S. chinensis*, which showed sensitivity toward Amphotericin B and Nystatin above a concentration of 100 μg/ml. In subsequent studies, Sheik et al. (2020) confirmed the role of *C. gloeosporioides* as an anticancer agent. Moreover, *C. gloeosporioides* is significantly involved in decolorizing carcinogenic dyes such as methylene blue and Congo red. Roopa et al. (2017) performed fungal endophyte diversity assessment on different species of *Salacia* (*S. chinensis*, *S. oblonga*, *S. fruticosa*, and *S. macrosperma*) and reported the *Salacia* spp. with rich endophytic microbes. The results confirmed the occurrence of 15 fungal endophytes such as *Alternaria alternata*, *Aspergillus niger*, *Cladosporium herbarum*, *Colletotrichum* spp., *Curvularia* spp., *Diaporthe perjuncta*, *Drechslera* spp., *Phoma* spp*.*, *Penicillium notatum*, *Myrothecium verrucaria*, *Gliocladium roseum*, *Fusarium oxysporium*, *Sterile* spp*.*, *Trichophyton mentagrophytes*, and *Xylaria* spp. from the stem of *S. chinensis*. This study also confirmed the occurrence of 12 fungal endophytes such as *Botryosphaeria rhodina*, *Cladosporium herbarum*, *Trichoderma longibrachiatum*, *Aspergillus terreus*, *A. niger*, *Fusarium oxysporium*, *Lasiodiplodia theobromae*, *Penicillium notatum*, *Phoma* spp*.*, *Sterile* spp*.*, *Coriolopsis caperata*, and *Pestalotiopsis* from the stem of *S. oblonga*. Ten different fungal endophytes, viz., *Drechslera* spp., *Curvularia* spp*.*, *Cladosporium herbarum*, *Pestalotiopsis* spp*.*, *Trichoderma longibrachiatum*, *Aspergillus terreus*, *Colletotrichum* spp*.*, *Diaporthe perjuncta*, *Myrothecium verrucaria*, and *Xylaria* spp. were isolated from the stem of *S. fruticosa* (Roopa et al. 2017). This study also identified nine fungal endophyes such as *Aspergillus terreus*, *A. niger*, *Phoma* spp*.*, *Cladosporium herbarum*, *Colletotrichum* spp*.*, *Penicillium notatum*, *Trichophyton mentagrophytes*, *Alternaria alternata*, and *Fusarium oxysporium* from the stem of *S. macrosperma*.

**Table 2:** Isolation, microbial classification, and modulatory role of endophytes in secondary metabolite production and elevating biological potential of *Salacia* spp

*Salacia* spp*.*	Source/plant part	Microbial classification	Microbial endophyte(s)	Function(s)	Reference
*S. chinensis*	Stem, leaf	Fungi	*Colletotrichum gloeosporioides*	Antifungal sensitivity	Bhagya et al. 2011
*S. chinensis*	Stem	Fungi	*Alternaria alternate*; *Aspergillus niger*; *Cladosporium herbarum*; *Colletotrichum* sp.; *Curvularia* sp.; *Diaporthe perjuncta*; *Drechslera* sp.; *Phoma* spp.; *Penicillium notatum*; *Myrothecium verrucaria*; *Gliocladium roseum*; *Fusarium oxysporum*; *Sterile* spp.; *Trichophyton mentagrophytes*; *Xylaria* spp.	NR	Roopa et al. 2017
*S. chinensis*	Stem, leaf	Fungi	*Colletotrichum gloeosporioides*	Potential source as anticancer agent. Significant role in decolorizing carcinogenic dyes such as methylene blue and Congo red	Sheik et al. 2020
*S. chinensis*	–	Bacteria	*Klebsiella* spp.; *K. variicola*; *Stenotrophomonas* spp.; *Enterobacter* spp.	NR	Webster et al. 2020
*S. chinensis*	Root, stem	Fungi	*Penicillium capsulatum*;* Aspergillus fumigatus*	Significantly modulates mangiferin biosynthesis	Kaur et al. 2022
*S. oblonga*	Root, stem	Fungi	*Botryosphaeria rhodina*; *Trichoderma longibrachiatum*; *Lasiodiplodia theobromae*; *Aspergillus niger*; *A. terreus*; *Coriolopsis caperata*; *Phomopsis* sp.;* Fusarium solani*	Significantly modulates taxol biosynthesis—an anticancer agent	Roopa et al. 2015
*S. oblonga*	Stem	Fungi	*Botryosphaeria rhodina*; *Cladosporium herbarum*; *Trichoderma longibrachiatum*; *Aspergillus terreus*; *A. niger*; *Fusarium oxysporum*; *Lasiodiplodia theobromae*; *Penicillium notatum*; *Phoma* spp.; *Sterile* spp.; *Coriolopsis caperata*;* Pestalotiopsis*	NR	Roopa et al. 2017
*S. oblonga*	Root, stem	Fungi	*Penicillium capsulatum*;* Aspergillus fumigatus*	Significantly modulates mangiferin biosynthesis	Kaur et al. 2022
*S. fruticosa*	Stem	Fungi	*Drechslera* spp.; *Curvularia* spp.; *Cladosporium herbarum*; *Pestalotiopsis* spp.; *Trichoderma longibrachiatum*; *Aspergillus terreus*; *Colletotrichum* spp. *Diaporthe perjuncta*; *Myrothecium verrucaria*; *Xylaria* spp.	NR	Roopa et al. 2017
*S. macrosperma*	Stem	Fungi	*Aspergillus terreus*; *A. niger*; *Phoma* spp.; *Cladosporium herbarum*; *Colletotrichum* spp.; *Penicillium notatum*; *Trichophyton mentagrophytes*; *Alternaria alternate*;* Fusarium oxysporum*	NR	Roopa et al. 2017
*S. macrosperma*		Bacteria	*Bacillus* spp*.*	NR	Webster et al. 2020
*S. reticulata*	Root, stem	Bacteria	*Bacillus licheniformis*; *B. subtilis*; *B. zanthoxyli*;* B. aryabhattai*	NR	Bhosale et al. 2021
*S. reticulata*	Root, stem	Fungi	*Aspergillus* sp.	NR	Bhosale et al. 2021
*S. chinensis*, *S. oblonga*, *S. fruticosa*, *S. macrosperma*	Stem	Fungi	*Fusarium oxysporum*, *Penicillium notatum*, *Pestalotiopsis* spp., *Phoma* spp., *Colletotrichum* spp. (dominant species)	Presence of steroids, tannins, sugars, proteins, flavonoids, saponins, terpenoids, and glycosides. Significantly enhances the antibacterial and anti-diabetic properties	Roopa et al. 2022

*NR* not reported

Webster et al. (2020) reported four bacterial endophytes such as *Klebsiella* spp., *K. variicola*, *Stenotrophomonas* spp., and *Enterobacter* spp. from *S. chinensis*; furthermore, this study also identified a fungal endophyte, *Bacillus* spp., in *S. macrosperma*. The fungal endophytes *Penicillium capsulatum* and *Aspergillus fumigatus* isolated from the stem and roots of *S. chinensis* and roots of *S. oblonga* significantly modulate the mangiferin biosynthesis (Kaur et al. 2022). Mangiferin is a naturally occurring multipotent xanthone that possesses numerous pharmacological properties and is involved in treating numerous human diseases and disorders (Chavan et al. 2015a; Feng et al. 2019). Eight fungal endophytes, i.e., *Botryosphaeria rhodina*, *Trichoderma longibrachiatum*, *Lasiodiplodia theobromae*, *Aspergillus niger*, *A. terreus*, *Coriolopsis caperata*, *Phomopsis* spp*.*, and *Fusarium solani*, have been isolated from the stem and roots of *S. oblonga*, where *Alternaria*, *Fusarium*, and *Aspergillus niger* significantly modulate taxol biosynthesis (Roopa et al. 2015). *Taxus brevifolia* and their endophytic microbes are the original sources of taxol, a widely used diterpenoid as a chemotherapy drug to treat lung, breast, and other types of cancer (Stierle et al. 1995). The stem and roots of *S. reticulata* also host four bacterial endophytes, such as *B. licheniformis*, *B. subtilis*, *B. zanthoxyli*, *B. aryabhattai*, and a fungal endophyte, i.e., *Aspergillus* spp. (Bhosale et al. 2021). Recently, Roopa et al. (2022) investigated different plant parts of four *Salacia* spp. (*S. chinensis*, *S. oblonga*, *S. fruticosa*, and *S. macrosperma*) for assessing endophyte diversity and their potential role in augmenting biological properties as well as the accumulation of secondary metabolites. The results confirmed that *F. oxysporum*, *Penicillium notatum*, *Pestalotiopsis* spp., *Phoma* spp., and *Colletotrichum* spp. have been found predominantly in *Salacia* spp. Moreover, these endophytes have shown potential anti-diabetic activity through alpha-amylase inhibitor activity and accumulate steroids, tannins, sugars, proteins, flavonoids, saponins, terpenoids, and glycosides at different levels.

The literature on the isolation and characterization of endophytes from *Salacia* spp. confirms the occurrence of fungal as well as bacterial microbes, which accumulate numerous secondary metabolites and significantly modulate the biosynthesis of marker compounds. These findings strongly suggest that the crude extracts and the phytochemicals isolated from the endophytic microbes of *Salacia* spp. can be industrialized as potent drug molecules in view of their pharmaceutical significance.

### Molecular markers for assessment and improvement of *Salacia* spp.

The advancement in DNA-based molecular markers has become a milestone in plant science research. These molecular markers are commonly practiced for the improvement of numerous plants, especially through plant breeding, genetic diversity analysis, population genetics, phylogenetics, mapping of mutations, assessment of genetic uniformity among tissue culture–raised plantlets, identifying adulteration in health-based foods, taxonomy and evolution, confirming the identity of individuals, etc. Literature on the utility of molecular markers confirmed their extensive role in genetic diversity assessment, phylogenetic studies, genetic integrity analysis of micropropagated plantlets, and authentication of health foods from *Salacia* spp (Table 3).

**Table 3:** Molecular interventions in *Salacia* spp

*Salacia* spp*.*	Goal	Molecular marker(s)	Outcome/observations	Reference
*S. pallescens*	Phylogenetics	*rbcL*	Phylogenetic analysis confirms two clades of order Celastrales. *Salacia* falls under second group which also consists of genera *Euonymus* and *Hippocratea*	Savolainen et al. 1994
*S. impressifolia* *S. nitida* *S. pallescens* *S. undulata*	Phylogenetics	26S nrDNA, Phytochrome B, *rbcL*,* atpB*	*Salacia*, *Catha*, *Maytenus*, and *Pristimera* are distinct and nested in the same group even after not being monophyletic genera	Simmons et al. 2001
*S. chinensis*	Genetic diversity	RAPD	RAPD marker found suitable for assessing genetic variation among *S. chinensis* populations	Mulye et al. 2013
*S. oblonga*	Genetic diversity	RAPD	Reported greatest genetic polymorphism between the *S. chinensis* samples	Maheswari et al. 2013
*S. chinensis*	Genetic stability analysis	RAPD, ISSR	Molecular markers confirmed over 99.66% genetic uniformity among in vitro–raised plantlets	Chavan et al. 2015b
*Salacia* spp.	Phylogenetics	ITS sequences of nrDNA	Morphological species demarcation did not corroborate with the ITS phylogeny	Devipriya et al. 2015
*S. oblonga*	Genomic mining	ITS	Discovery of novel taxol-producing endophytic fungi	Roopa et al. 2015
*S. reticulata*	Genetic diversity	RAPD	Extent of polymorphism observed among the accessions of *S. reticulata*	Dhanasri et al. 2015
*Salacia* spp.	Species discrimination	ITS2, *trnH-psbA*, *matK*,* rbcL*	ITS2 barcode discriminate analyzed *Salacia* spp. (*S. beddomei*, *S. chinensis*, *S. fruticosa*, *S. macrosperma*, *S. malabarica*, *S. oblonga* var. *oblonga*, *S. oblonga* var. *kakkayamana*, *S. vellaniana*, and *S. agasthiamalana*) with 100% accuracy	Dev et al. 2015
*S. chinensis*	Genetic stability analysis	FRAPD	5% genetic variation detected	Majid et al. 2018
*S. chinensis* *S. macrosperma* *S. fruticosa* *S. oblonga*	Genetic diversity and phylogenetic relationship	RAPD, ISSR, ITS	RAPD (41.45 ± 10%), ISSR (33.58 ± 6.52%), and ITS (25.50 ± 17.25%) revealed significant variance within and among the *Salacia* spp.	Bajpe et al. 2018
*S. amplifolia*	Complete plastome sequence	–	The analysis confirms the complete plastome is 163,255 bp in length which contains 113 genes. 79 are unique protein-coding genes, 30 unique tRNA genes, and 4 unique rRNA genes	Lin et al. 2019
*Salacia* spp.	Species variability	RAPD	*Salacia chinensis* is very distinct from other species such as *S. beddomei*, *S. fruticosa*, *S. gambleana*, *S. macrosperma*, *S. malabarica*, and* S. oblonga*	Priya et al. 2019
*Salacia* spp*.*	Phylogenetics	ITS 2 secondary structure	Phylogenetic trees constructed based on sequence and structural features of ITS 2 confirmed the two evolutionary lines. One line leads to the present-day *S. chinensis* and the other line further diversified and led to the rest of the *Salacia* spp. i.e., *S. agasthiamalana*, *S. brunoniana*, *S. fruticosa*, *S. gambleana*, *S. oblonga*, *S. malabarica*, and* S. wayanadica*	Muhammad Anaz et al. 2021
*S. reticulata* *S. oblonga* *S. chinensis*	Genetic identification, confirmation of food adulteration	nrDNA ITS sequences, RFLP	Useful information to authenticate *Salacia* species and results confirm that *S. chinensis* and *S. reticulata* are the major sources of commercially available *Salacia*-derived food products	Zhu et al. 2021
*S. chinensis* *S. fruticosa* *S. macrosperma* *S. oblonga*	Genetic diversity, species authentication	ISSR, *rbcL*, *matK*, ITS	ISSR primers revealed 29.21 ± 7.89% of polymorphism among *Salacia* spp. and nine other genera of family Celastraceae. DNA barcodes using *matK*, *rbcL* contributed correct identification of species	Badiger et al. 2021

*FRAPD* fluorescently labeled random amplified polymorphic DNA; *ISSR* inter simple sequence repeats, *ITS* internal transcribed spacer, *nrDNA* nuclear ribosomal DNA; *RAPD* random amplified polymorphic DNA; *rbcL* ribulose-bisphosphate carboxylase gene, *RFLP* restriction fragment length polymorphism

The first attempt at the integration of molecular markers in the genus *Salacia* was by Savolainen et al. (1994). The phylogenetic analysis of genera falling under order Celastrales through *rbcL* confirmed the familiarity of genus *Salacia* with genera *Euonymus* and *Hippocratea*. In subsequent studies on phylogenetics, 26S nrDNA, phytochrome B, *rbcL*, and *atpB* nested four *Salacia* spp. (*S. impressifolia*, *S. nitida*, *S. pallescens*, and *S. undulata*) with close genera such as *Catha*, *Maytenus*, and *Pristimera* (Simmons et al. 2001). The Indian clade of *Salacia* has been characterized through the nuclear ribosomal DNA (nrDNA) internal transcribed spacer (ITS 1 and ITS 2) region for establishing the relationship among species (Devipriya et al. 2015). The investigation of a total of 24 species of the genus *Salacia* (8 onsite collection and 16 through GenBank accession), 6 allied genera, and 2 outgroups showed divergence among species, and it was also confirmed that the morphological species demarcation did not corroborate with the ITS phylogeny. In further studies, Dev et al. (2015) confirmed the species discrimination through ITS2, *trnH-psbA*, *matK*, and *rbcL* across one variety and eight species of genus *Salacia*, including *S. beddomei*, *S. chinensis*, *S. fruticosa*, *S. macrosperma*, *S. malabarica*, *S. oblonga* var. *oblonga*, *S. oblonga* var. *kakkayamana*, *S. vellaniana*, and *S. agasthiamalana* with 100% accuracy. Alongside the ITS marker, RAPD and ISSR also significantly contributed toward assessing genetic diversity and establishing possible relationships among *Salacia* spp., viz., *S. chinensis*, *S. macrosperma*, *S. fruticosa*, and *S. oblonga* (Bajpe et al. 2018). The molecular fingerprints confirmed the polymorphism revealed by RAPD (41.45 ± 10%), ISSR (33.58 ± 6.52%), and ITS (25.50 ± 17.25%), which revealed significant variance within and among the *Salacia* spp. Recently, ITS-2 secondary structure-based molecular characterization has been done for construction phylogeny of eight *Salacia* spp. (Anaz et al. 2021). The phylogenetic tree based on the sequence and structural features of ITS 2 confirmed the two evolutionary lines among *Salacia* spp. One line leads to the present-day *S. chinensis*, and the other line further diversifies and leads to the rest of the *Salacia* spp., i.e., *S. agasthiamalana*, *S. brunoniana*, *S. fruticosa*, *S. gambleana*, *S. oblonga*, *S. malabarica*, and *S. wayanadica*.

RAPD and ISSR markers contributed significantly to detect genetic divergence within and among the *Salacia* spp. The RAPD marker was found suitable for the assessment of genetic variation among different populations of *S. chinensis* (Mulye et al. 2013). Further investigations on RAPD analysis reported the greatest genetic polymorphism among different samples of *S. oblonga* (Maheswari et al. 2013). Different accessions of *S. reticulata* were also analyzed using RAPD markers, and the results also supported the previous findings with the extent of polymorphism among the accessions (Dhanasri et al. 2015). In subsequent studies, Priya et al. (2019) also detected genetic variability within and among the eight *Salacia* spp. using RAPD markers. The results also confirmed the distinctiveness of *S. chinensis* from other species such as *S. beddomei*, *S. fruticosa*, *S. gambleana*, *S. macrosperma*, *S. malabarica*, and *S. oblonga.* In another study, an average of 29.21 ± 7.89% of polymorphism among four *Salacia* spp. such as *S. chinensis*, *S. fruticosa*, *S. macrosperma*, and *S. oblonga* was detected by ISSR markers (Badiger et al. 2021).

DNA barcoding is the most effective and emerging method of molecular identification of flowering plants as well as plant-based health foods (Kshirsagar et al. 2017; Anaz et al. 2021). A literature survey confirmed that DNA barcodes have been developed for authentication and accurate identification of several *Salacia* spp. Badiger et al. (2021) developed barcodes using *rbcL*, *matK*, and ITS markers for accurate identification of four *Salacia* species, viz., *S. chinensis*, *S. fruticosa*, *S. macrosperma*, and *S. oblonga*. In another study, Zhu et al. (2021) employed nrDNA ITS sequences and RFLP markers for genetic identification and confirmation of *Salacia-*based health food authentication. The markers found useful to authenticate *Salacia* spp., and the results also confirmed that *S. chinensis* and *S. reticulata* are the chief sources of commercially existing *Salacia*-derived food products.

**Fig. 5:**
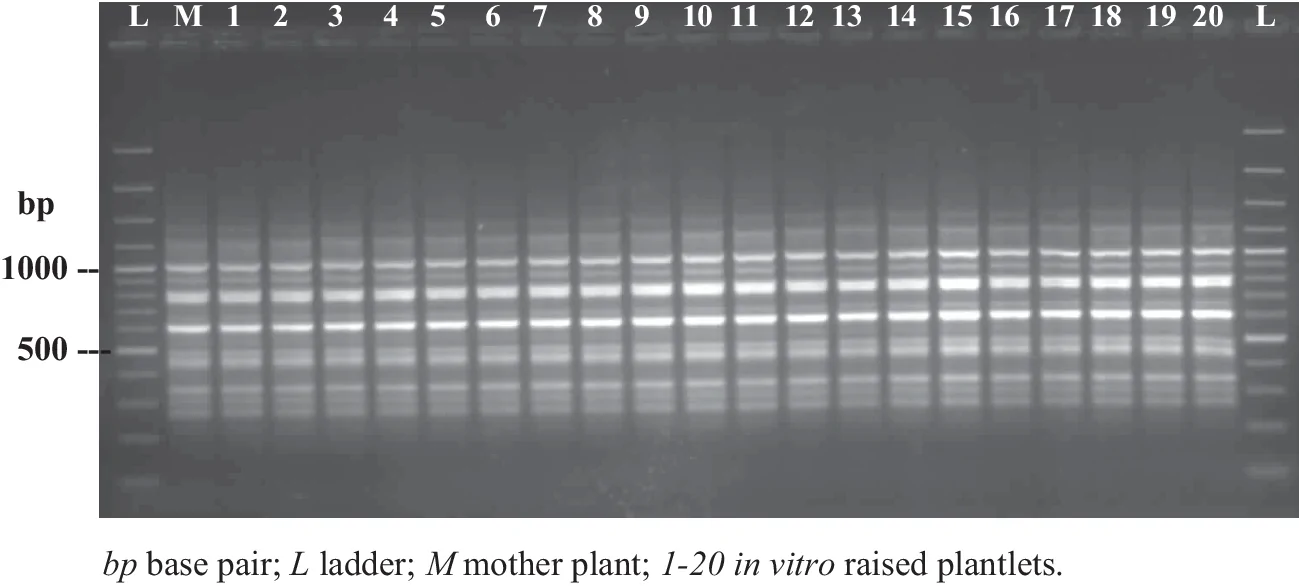
Genetic fidelity assessment of in vitro*–*raised plantlets of *S. chinensis* by ISSR analysis (UBC: 801)

Confirmation of genetic integrity among tissue culture–raised clones of ornamental, medicinally important, and agricultural crop plants is the key stage before their large-scale propagation and commercialization. Molecular markers including RAPD, RFLP, AFLP, FRAPD, SSR, ISSR, SSAP, and SCoT have been regularly utilized to confirm the genetic integrity of tissue culture–regenerated plantlets of numerous flowering plants (Chavan et al. 2015b; Bhattacharyya et al., 2013; Majid et al. 2018). In *Salacia*, few reports focused on the genetic stability analysis of tissue-culture plantlets. Chavan et al. (2015b) confirmed the genetic uniformity of in vitro propagated plantlets of *S. chinensis* using ISSR and RAPD markers. Combined, 3871 bands were produced by 17 ISSR and 12 RAPD primers across a mother plant and 20 micropropagated plantlets, wherein 3858 (99.66%) bands showed similarity among tested plantlets and the rest of the bands showed divergence. The high level of similarity confirmed the genetic homogeneity of the micropropagated clones of *S. chinensis* (Fig. 5). Another report on *S. chinensis* detected 5% variation among donor and in vitro–propagated plantlets as assessed by FRAPD (Majid et al. 2018).

Complete plastome sequencing is a vital intervention for knowing the complete genetic make-up of organisms which supports policy design, especially conservation and improvement of plants. Lin et al. (2019) performed the complete plastome sequencing for *S. amplifolia*, which is the only report available for the genus *Salacia*. The analysis confirmed the size of the complete plastome (163,255 bp in length) which contains 113 genes, of which 79 are unique protein-coding genes, 30 are unique tRNA genes, and 4 are unique rRNA genes. The utility of molecular markers in microbial endophyte improvement is well established in flowering plants. Only a report by Roopa et al. (2015) addressed the ITS-based genome mining approach for the discovery of novel taxol-producing endophytic fungi from *S. oblonga*.

RAPD, ISSR, and FRAPD are routinely practiced for the genetic diversity analysis and confirmation of genetic integrity among tissue culture–raised plantlets of *Salacia* spp. However, ITS, phytochrome B, *rbcL*, *atpB*, *trnH-psbA*, and *matK* are among the commonly used markers for establishing phylogeny and developing barcodes for the precise authentication of species and identifying *Salacia*-based health food adulterations.

### Salacia-based nanomaterials in enhancing biological properties

Plant-based nanomaterials are routinely used to elevate secondary metabolites as well as the biological properties of several plant species. Such biogenic nanomaterials have advantages over other nanomaterials because they are biodegradable, biocompatible, and generally recognized as safe (Patil and Chandrasekaran 2020). Different types of nanomaterials have been fabricated from various organs of *Salacia* spp. The first attempt at the synthesis, characterization, and analysis of biological potential was by Jadhav et al. (2015) for *S. chinensis*. The results confirmed the antibacterial potential of silver nanoparticles synthesized from bark, which were found to be effective against *Staphylococcus aureus* and *Pseudomonas aeruginosa*. The aqueous leaf extract of *S. chinensis* have been used for synthesis of silver and copper nanomaterials (Chavan and Ghadage 2018). Both the nanoparticles have potential antibacterial properties against *Bacillus subtilis*, *Escherichia coli*,* S. aureus*, and* P. aeruginosa* that validated the action of these nanoparticles against these infectious microbes. In further studies in *S. chinensis*, the silver nanoparticles have the potential to enhance antioxidant and antimicrobial potential (Abhijit et al. 2019). The synthesized nanoparticles were assessed for their antioxidant potential using a DPPH assay, and it was found that nanoparticles have potent scavenging activity over crude extract. Moreover, the antimicrobial potential of the particles was tested against pathogenic fungi and bacteria, wherein nanomaterials showed moderate growth inhibition on *E. coli*, *Candida albicans*, and *C. tropicalis*. Recently, Nagesh et al. (2022) synthesized silver nanomaterials from the roots of *S. chinensis*, which is able to modulate biological properties such as antibacterial, antifungal, and antiproliferative effects. The leaf extract–derived silver nanoparticles from *S. mulbarica* proved to be a better antibacterial agent against *E. coli* and *B. subtilis* and also possess the capability of ct-DNA damage via releasing of reactive oxygen species (Espenti et al. 2020). In recent times, environmentally benign silver bio-nanomaterials have been synthesized from the root extracts of *S. oblonga* and served as potent antioxidant, antibacterial, and anti-diabetic agents (Dugganaboyana et al. 2023). Recently, Sabeena et al. (2023) performed a comparative study on chemical and green approaches for synthesis of nanocomposites from leaf extracts of *S. reticulata* and subsequently characterized for in vivo and in vitro biological properties. The green synthesized nanocomposites, i.e., iron oxide, cerium oxide, titanium dioxide, silica gel, and chitosan, of *S. reticulata* possesses strong anti-inflammatory, antibacterial, and anti-diabetic properties, as well as considerable suppression of high activation in in vivo zebrafish embryo toxicity (Sabeena et al. 2023).

Among the metal nanoparticles, gold nanoparticles are recognized as the most potent, biocompatible, and environment friendly (Khan et al. 2019). The first attempt at the synthesis and characterization of nanoparticles (gold) in the genus *Salacia* was by Jadhav et al. (2018). The gold nanoparticles synthesized from the bark of *S. chinensis* were evaluated for their osteoinductive potential for application in implant dentistry. The results confirmed that the stable, biocompatible, and eco-friendly gold nanoparticles can be used as an effective bone inductive agent during dental implant therapy. The gold nanoparticles have been synthesized using leaf extracts of *S. fruticosa*, which have antibacterial activity against the bacterial pathogens such as *S. aureus* and *P. aeruginosa* (Keshavamurthy and Ravishankar Rai 2021). The results on green nanomaterial synthesis using different plant organs of *Salacia* spp. are mainly through silver, copper, gold, iron, cerium, titanium, silica, and chitosan nanoparticles. These nanomaterials proved their role in elevating the biological potential of *Salacia* spp. Moreover, these green synthesized nanomaterials are more affordable, environmentally friendly, and biocompatible than chemically synthesized ones.

## Conclusion and future perspectives

In conclusion, the in vitro biotechnology of *Salacia* spp. is mainly focused on in vitro regeneration and secondary metabolite production. Nodal segments are among the commonly used explants for initiating the cultures, while leaves served better for initiating callus cultures. MS medium is the exclusive requirement during various in vitro stages alongside diverse types and concentrations of PGRs, especially BAP and either IBA or IAA for shoot initiation and multiplication in most of the *Salacia* spp. IBA supported in vitro rooting in almost all *Salacia* spp., while a combination of BAP and 2,4-D served better for induction of callus. Different in vitro regeneration stages and the elicitation of various cultures significantly enhance the accumulation of secondary metabolites. Moreover, microbial endophytes and nanomaterials (silver, copper, and gold) also up-scale the biochemical marker compounds and bio-efficacy of different *Salacia* spp. Molecular markers found suitable for assessing genetic diversity among and between the species (ITS, 26S nrDNA, Phytochrome B, rbcL, atpB, trnH-psbA, matK, RAPD, ISSR) confirmed the genetic stability of tissue culture–raised plantlets (RAPD, FRAPD, and ISSR), as well as authentication and identification of *Salacia* spp. (ISSR, rbcL, matK, ITS). Moreover, it serves as an authentic tool for confirmation of adulteration in *Salacia*-based health foods (nrDNA ITS sequences, RFLP). There are several untouched areas of research on in vitro biotechnology of *Salacia* spp., such as synthetic seed production, cryopreservation, agrobacterium-mediated genetic transformation, and omics, that deserve special attention to advance the biotechnology of *Salacia* spp.

## Data Availability

All data generated or analyzed during this study are included in the submitted manuscript.
